# Supplementary material for: Taf14 recognizes a common motif in transcriptional machineries and facilitates their clustering by phase separation
Source: Nat Commun. 2020 Aug 21;11:4206. doi: 10.1038/s41467-020-18021-7 (PMC7442819; doi:10.1038/s41467-020-18021-7)
Supplement: Supplementary file 1 — Supplementary Information [file 41467_2020_18021_MOESM1_ESM.pdf]

Supplementary Information

Taf14 recognizes a common motif in transcriptional machineries and facilitates their clustering by phase separation

Guochao Chen, Duo Wang, Bin Wu et al.

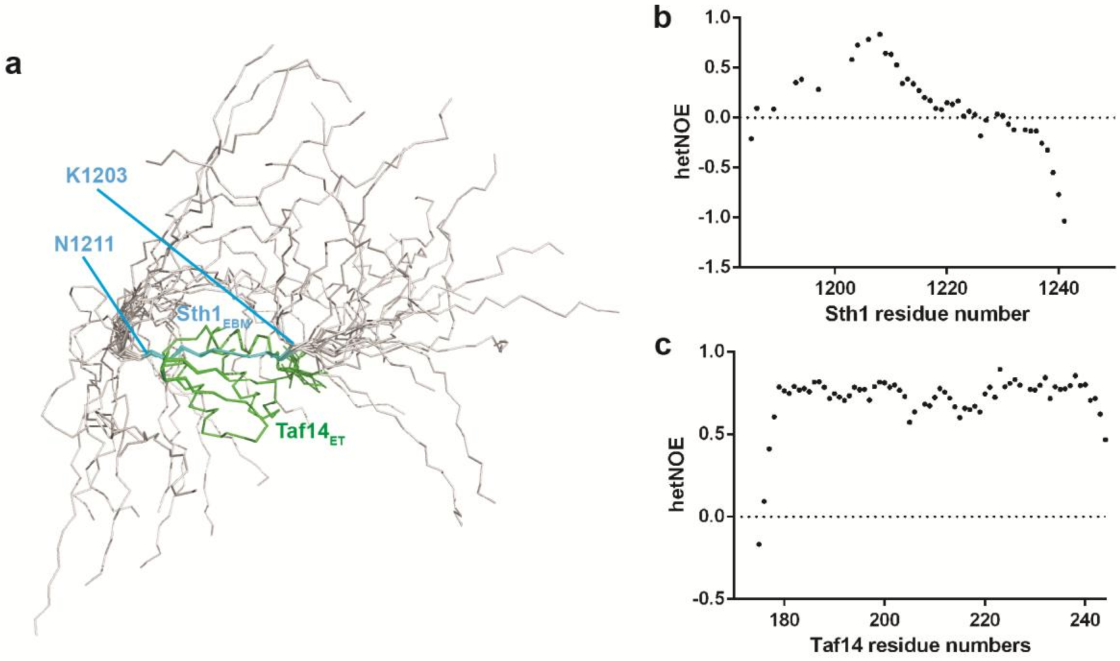

Supplementary Figure 1. NMR analyses of $Taf14_{ET}$-$Sth1_{EBM}$ complex

a. The ensemble of the 20 lowest energy conformers of the $Taf14_{ET}$-$Sth1_{EBM}$ complex. $Taf14_{ET}$ is shown in green. The core regions of $Sth1_{EBM}$ converge from K1203 to N1211. Other parts of $Sth1_{EBM}$ are flexible.

b. Heteronuclear NOE values measured at 25 ℃ for ^{15}N-labeled $Sth1_{EBM}$ in complex with unlabeled $Taf14_{ET}$.

c. Heteronuclear NOE values measured at 25 ℃ for ^{15}N-labeled $Taf14_{ET}$ in complex with unlabeled $Sth1_{EBM}$.

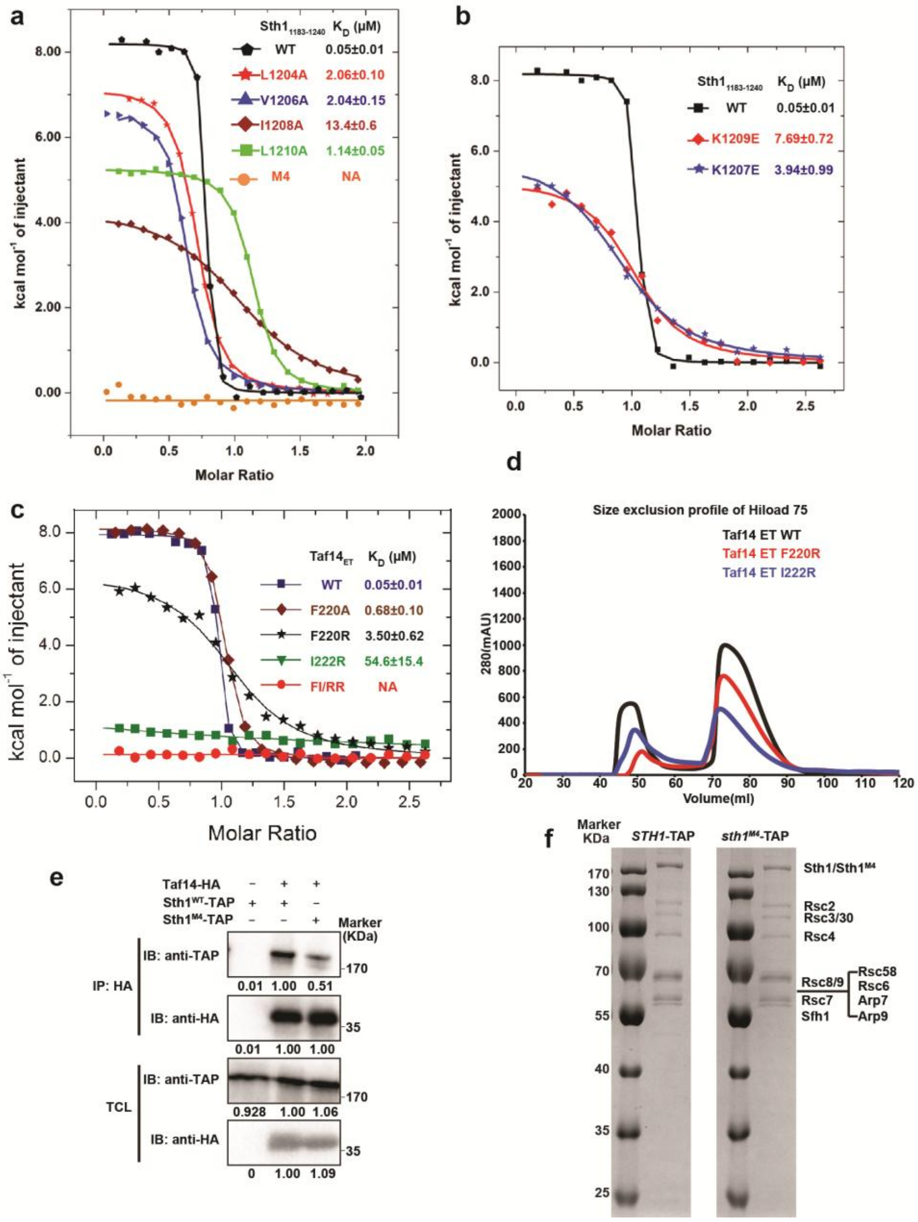

Supplementary Figure 2. Mutation analyses of Taf14-Sth1 complex.

a. ITC assays show that mutations of hydrophobic residues of Sth1 decrease the interaction between $Taf14_{ET}$ and $Sth1_{EBM}$.

b. ITC assays show that mutations of positively charged residues of Sth1 decrease the interaction between $Taf14_{ET}$ and $Sth1_{EBM}$.

c. ITC assays show that mutations of hydrophobic residues of Taf14 decrease the interaction between $Taf14_{ET}$ and $Sth1_{EBM}$.

d. Gel-filtration profiles of $Taf14_{ET}$ mutations showed that these mutations did not affect the overall structural integrity of $Taf14_{ET}$.

e. Another batch of co-immunoprecipitation experiment using an HA antibody showed that $Sth1^{M4}$ mutation weakened the interaction with Taf14. Taf14 was tagged with HA; Sth1 was tagged with TAP. TCL, total cell lysate; IP, immune-precipitation; IB, immune-blot.

f. TAP purification shows that *sth1*^{M4} mutation does not affect the integrity of the RSC complex.

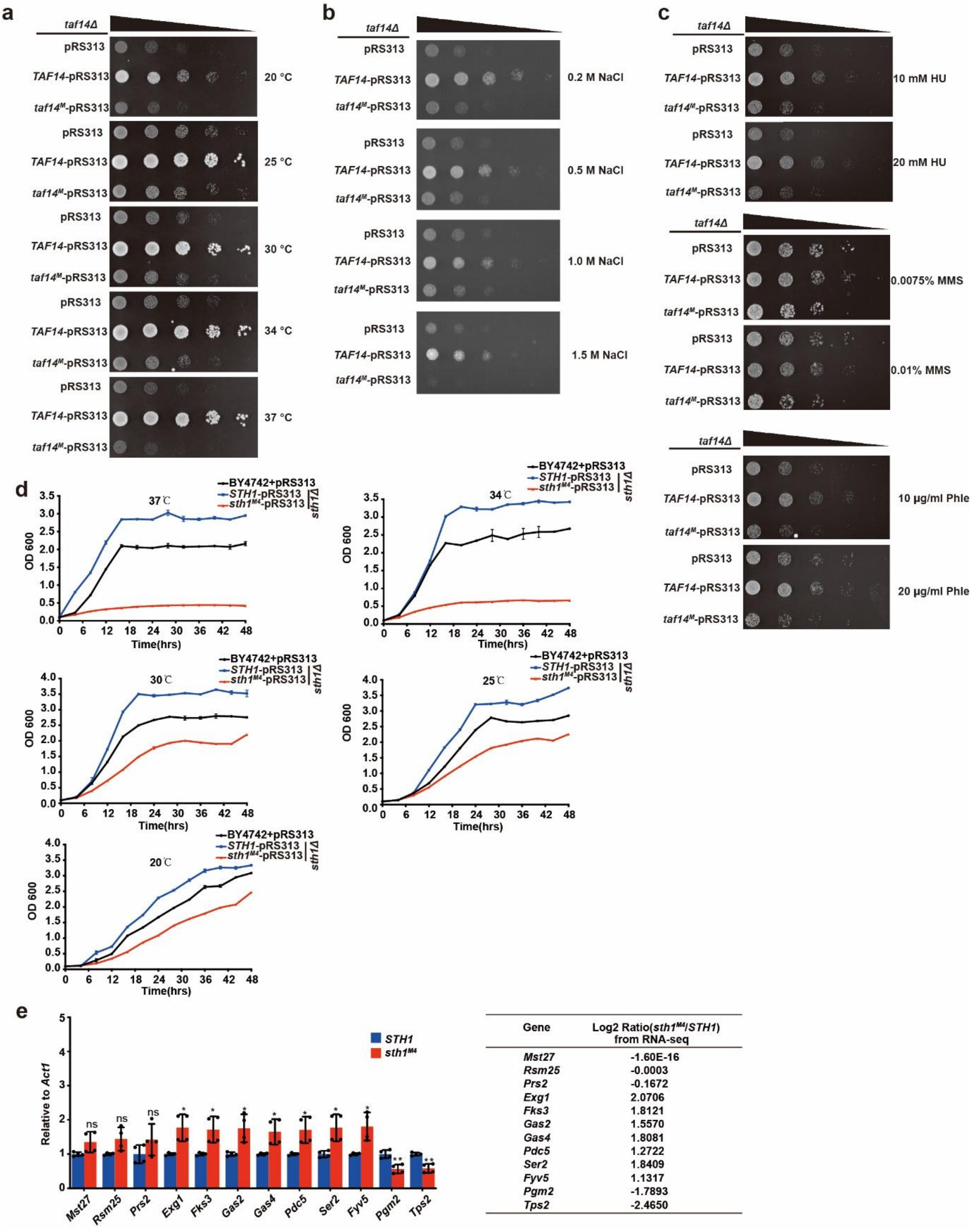

Gene	Log2 Ratio(*sth1*^{M4}/*STH1*) from RNA-seq
Mst27	-1.60E-16
Rsm25	-0.0003
Prs2	-0.1672
Exg1	2.0706
Fks3	1.8121
Gas2	1.5570
Gas4	1.8081
Pdc5	1.2722
Ser2	1.8409
Fyv5	1.1317
Pgm2	-1.7893
Tps2	-2.4650

Supplementary Figure 3. Functional analyses of Taf14 and Sth1 mutations in yeast cells.

a. Spotting assays with *taf14Δ* and *taf14^M* strains compared with WT strains at various temperatures. The *taf14^M* is Taf14 F220R/I222R mutation that disrupts $Sth1_{EBM}$ binding. The *taf14Δ* and *taf14^M* strains showed growth defects at all the temperatures.

b. Spotting assays with *taf14Δ* and *taf14^M* strains compared with WT strains on plates containing different NaCl concentrations at 30 ℃. The *taf14Δ* and *taf14^M* strains showed growth defects at all the NaCl concentrations.

c. Spotting assays with *taf14Δ* and *taf14^M* strains compared with WT strains on plates containing indicated DNA-damage agents at 30 ℃.

d. Growth curves at *STH1* and *sth1^{M4}* at different temperatures. The *sth1^{M4}* mutant showed much delayed growth when the temperature was above 30 ℃, but had similar (albeit slightly slow) growth rate compared to the WT strains. Data are presented as mean ± SD, n = 3.

e. The qPCR validation of the RNA-seq results. The change levels from RNA-seq were shown. Error bars represent standard deviations of four replicates. The relative expression level of wild-type *STH1* strain genes was set to 1. $*P < 0.05$; $**P < 0.01$; $***P < 0.001$; $****P < 0.0001$. Two-tailed Student's *t*-test. Data are presented as mean ± SD, n = 4.

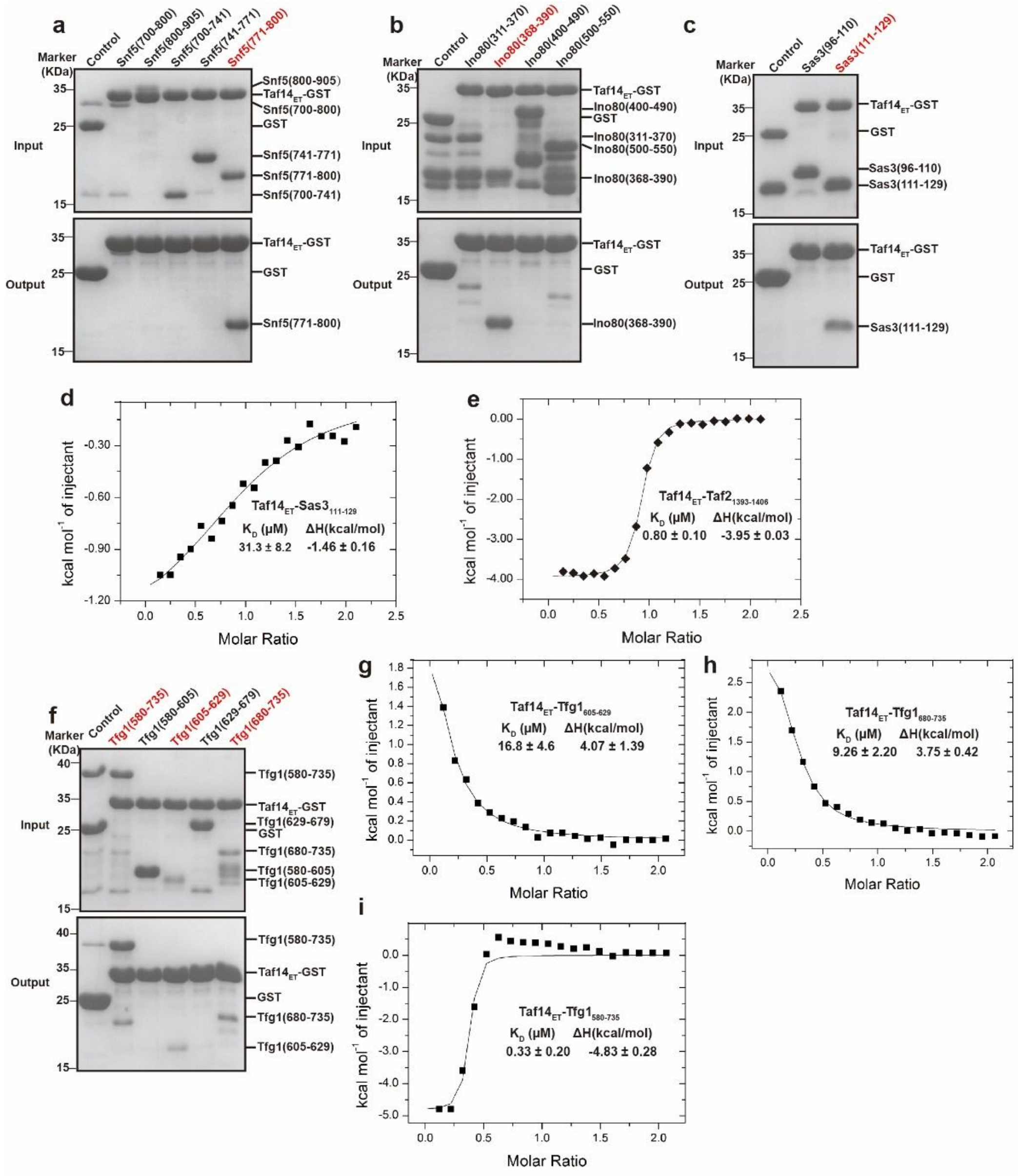

Supplementary Figure 4. GST pull-down assays to map the Taf14$_{ET}$-binding fragments.

a. Mapping the Taf14$_{ET}$-interaction regions of Snf5. Snf5$_{771\text{-}800}$ is pulled down by GST-Taf14$_{ET}$. All the Snf5 proteins are Sumo-fusion proteins to increase the visibility of the short peptides on SDS-PAGE. The Snf5 fragment binding Taf14$_{ET}$ was labeled red. The input controls were shown as 10% of the total proteins used to pull-down assays.

b. Mapping the Taf14$_{ET}$-interaction regions of Ino80. Ino80$_{368\text{-}390}$ has the strongest interaction with GST-Taf14$_{ET}$. All the Ino80 proteins are Sumo-fusion proteins to

increase the visibility of the short peptides on SDS-PAGE. The Ino80 fragment binding $Taf14_{ET}$ was labeled red.

c. Mapping the $Taf14_{ET}$-interaction regions of Sas3. $Sas3_{111-129}$ is pulled down by GST-$Taf14_{ET}$. All the Sas3 proteins are Sumo-fusion proteins to increase the visibility of the short peptides on SDS-PAGE. The Sas3 fragment binding $Taf14_{ET}$ was labeled red.

d. The ITC plot for $Sas3_{111-129}$ (1 mM) titrated into $Taf14_{ET}$ (0.1 mM). The dissociation constant (K_D), enthalpy change (ΔH), and their fitting errors from this ITC plot were shown.

e. The ITC plot for $Taf2_{1393-1406}$ (1 mM) titrated into $Taf14_{ET}$ (0.1 mM).

f. Mapping the $Taf14_{ET}$-interaction regions of Tfg1. $Tfg1_{605-629}$ and $Tfg1_{680-735}$ have weak interactions with GST-$Taf14_{ET}$, while $Tfg1_{580-735}$ has a much stronger interaction with GST-$Taf14_{ET}$. All the Tfg1 proteins are Sumo-fusion proteins to increase the visibility of the short peptides on SDS-PAGE. The Tfg1 fragments binding $Taf14_{ET}$ were labeled red.

g. The ITC plot for $Tfg1_{605-629}$ (1 mM) titrated into $Taf14_{ET}$ (0.1 mM).

h. The ITC plot for $Tfg1_{680-735}$ (1 mM) titrated into $Taf14_{ET}$ (0.1 mM).

i. The ITC plot for $Tfg1_{580-735}$ (1 mM) titrated into $Taf14_{ET}$ (0.1 mM).

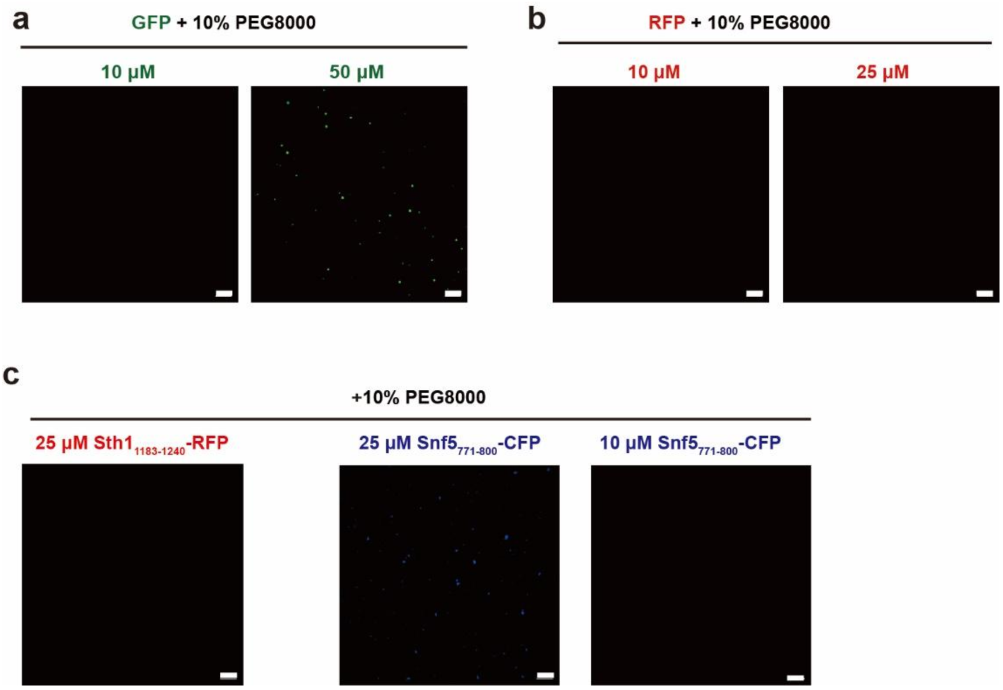

Supplementary Figure 5. Phase separation of some control proteins.

a. Representative images of GFP at two different concentrations in the droplet formation buffer (25 mM Tris-HCl, pH 8.0, 150 mM NaCl, 10% PEG8000). GFP alone formed small droplets at 50 μM concentration, but not at 25 (Fig.5a) or 10 μM, so we kept the protein concentration below 25 μM in the droplet formation assays.

b. Representative images of RFP at two different concentrations in the droplet formation buffer (25 mM Tris-HCl, pH 8.0, 150 mM NaCl, 10% PEG8000). At these concentrations, RFP did not form any droplet.

c. Representative images of Sth1$_{1183-1240}$-RFP and Snf5$_{771-800}$ -CFP in the same droplet formation buffer. Sth1$_{1183-1240}$-RFP did not form any droplet at 25 μM concentration. It should be noted that Snf5$_{771-800}$–CFP did not form any droplet at 10 μM concentration but formed small non-spherical aggregates at 25 μM concentration. So in the droplet formation assays with Snf5$_{771-800}$–CFP, the protein concentration was kept below 10 μM.

Supplementary Table 1. ***Saccharomyces cerevisiae*** **strains used in this study.**

Names of strains	Genotype
Fig.2e and Supplementary Fig. 2e, 2f	
BY4742	*MATα his3Δ1 leu2Δ0 lys2Δ0 ura3Δ0*
BY4742-1 (*TAF14-HA*)	BY4742 *TAF14-3HA::KanMX6*
BY4742-2(*STH1-TAP/TAF14-HA*)	BY4742 *STH1-TAP::URA3,TAF14-3HA ::KanMX6*
BY4742-2-Cas9 (*STH1-TAP/TAF14-HA*)	BY4742 *STH1-TAP::URA3,TAF14-3HA::KanMX6*, pCas9
BY4742-3 (*sth1*^{M4}*-TAP/TAF14-HA*)	BY4742 *sth1*^{M4}*-TAP::URA3,TAF14-3HA::KanMX6*
Fig. 3a-c, Supplementary Fig. 3d	
BY4742-4 (pRS313)	BY4742, pRS313
BY4742-5 (*STH1*-pRS313)	BY haploid *sth1Δ :: KanMX4*, pRS313-*STH1*
BY4742-6 (*sth1*^{M4}-pRS313)	BY haploid *sth1Δ :: KanMX4*, pRS313-*sth1*^{M4}
Supplementary Figure 3a-c	
BY4742-7 (pRS313)	BY4742 *taf14Δ :: KanMX4*, pRS313
BY4742-8 (*TAF14*-pRS313)	BY4742 *taf14Δ :: KanMX4*, pRS313-*TAF14*
BY4742-9 (*taf14*^{M}-pRS313)	BY4742 *taf14Δ :: KanMX4*, pRS313-*taf14*^{M}

Supplementary Table 2. Plasmids used in this study.

Names of plasmids	Purpose
Fig. 1	
pet28b-Sth1$_{(1183-1359)}$	Purification of Sth1 fragments 1183-1259
pet28b-Sth1$_{(1183-1240)}$	Purification of Sth1 fragments 1183-1240
pet28b-Sth1$_{(1248-1359)}$	Purification of Sth1 fragments 1248-1359
pet28b-Sth1$_{(1199-1225)}$	Purification of Sth1 fragments 1199-1225
pGEX6P-1-Taf14$_{174-244}$	Purification of Taf14 ET domain
Fig. 2 and Supplementary Fig. 2	
pet28b-Sth1$_{(1183-1240)}$ L1204A	Purification of Sth1$_{EBM}$ mutant L1204A
pet28b-Sth1$_{(1183-1240)}$ V1206A	Purification of Sth1$_{EBM}$ mutant V1206A
pet28b-Sth1$_{(1183-1240)}$ I1208A	Purification of Sth1$_{EBM}$ mutant I1208A
pet28b-Sth1$_{(1183-1240)}$ L1210A	Purification of Sth1$_{EBM}$ mutant L1210A
pet28b-Sth1$_{(1183-1240)}^{M4}$	Purification of Sth1$_{EBM}^{L1204A/V1206A/I1208A/L1210A}$
pet28b-Sth1$_{(1183-1240)}$ K1207E	Purification of Sth1$_{EBM}$ mutant K1207E
pet28b-Sth1$_{(1183-1240)}$ K1209E	Purification of Sth1$_{EBM}$ mutant K1209E
pGEX6P-1-Taf14$_{174-244}$ F220A	Purification of Taf14$_{ET}$ mutant F220A
pGEX6P-1-Taf14$_{174-244}$ F220R	Purification of Taf14$_{ET}$ mutant F220R
pGEX6P-1-Taf14$_{174-244}$ I222R	Purification of Taf14$_{ET}$ mutant I222R
pGEX6P-1-Taf14$_{174-244}$ F220R/I222R	Purification of Taf14$_{ET}$ mutant F220R/I222R
Fig. 4 and Supplementary Fig. 4	
pet28b-Sth1$_{(700-800)}$	Purification of Snf5 fragments with Sumo tag
pet28b-Snf5$_{(800-905)}$	Purification of Snf5 fragments with Sumo tag
pet28b-Snf5$_{(700-741)}$	Purification of Snf5 fragments with Sumo tag
pet28b-Snf5$_{(771-800)}$	Purification of Snf5 fragments with Sumo tag
pet28b-Ino80$_{(311-370)}$	Purification of Ino80 fragments with Sumo tag
pet28b-Ino80$_{(368-390)}$	Purification of Ino80 fragments with Sumo tag
pet28b-Ino80$_{(400-490)}$	Purification of Ino80 fragments with Sumo tag
pet28b-Ino80$_{(500-550)}$	Purification of Ino80 fragments with Sumo tag
pet28b-Sas3$_{(96-110)}$	Purification of Sas3 fragments with Sumo tag
pet28b-Sas3$_{(111-129)}$	Purification of Sas3 fragments with Sumo tag
pet28b-Tfg1$_{(580-735)}$	Purification of Tfg1 fragments with Sumo tag
pet28b-Tfg1$_{(580-605)}$	Purification of Tfg1 fragments with Sumo tag
pet28b-Tfg1$_{(629-679)}$	Purification of Tfg1 fragments with Sumo tag
pet28b-Tfg1$_{(680-735)}$	Purification of Tfg1 fragments with Sumo tag
Fig. 5 and Supplementary Fig. 5	
pet28b-GFP	Purification of GFP
pet28b-RFP	Purification of RFP
pet28b-CFP	Purification of CFP
pet28b-Taf14$_{174-244}$-GFP	Purification of Taf14$_{ET}$ with C-terminal GFP
pet28b-Sth1$_{(1183-1240)}$-RFP	Purification of Sth1$_{EBM}$ with C-terminal RFP
pet28b-Snf5$_{(771-800)}$-CFP	Purification of Snf5$_{EBM}$ with C-terminal CFP
Fig. 6	
pGEX6P-1-Bdf1$_{525-595}$	Purification of Bdf1 ET domain with GST tag
pGEX6P-1-Bdf2$_{514-585}$	Purification of Bdf2 ET domain with GST tag
pGEX6P-1-AF9$_{490-568}$	Purification of AF9 ET domain with GST tag
pet28b-AF9$_{490-568}$-GFP	Purification of AF9$_{ET}$ with C-terminal GFP
pet28b-BRD$_{601-683}$-GFP	Purification of BRD4$_{ET}$ with C-terminal GFP

Supplementary Table 3. Primers used in this study.

**Names of constructs**	**primer sequence 5'-3'**
Fig. 1	
Sth1$_{(1183\text{-}1359)}$	F:GCGCGGATCCGAAGTGAAAAGCTCTAGTGTTG<br>R: GCGCCTCGAGTTATGAGGAGTGTTCTTTAAACCATTC
Sth1$_{(1183\text{-}1240)}$	F: GCGCGGATCCGAAGTGAAAAGCTCTAGTGTTG<br>R:GCGCCTCGAGTTACTTCGCAGCGGTTTTCTTCGCC
Sth1$_{(1248\text{-}1359)}$	F: GCGCAGATCTTCGCTTGGGATTTTTCCCACG<br>R:GCGCCTCGAGTTACGAAGAGTGTTCCTTGAACCATTCATCAG TAAACTCATTTAACTTGTCAGCATCAACGTAAACCCAGGAGCCC TC
Sth1$_{(1199\text{-}1225)}$	F: GCGCGGATCCAAGAAAAAACCGAAACTGACCG<br>R:GCCGCTCGAGTTAATATTCAGCGCGTTTACCATCATTGTTTTC CAG
Taf14$_{(174\text{-}244)}$	F: GCGCGGATCCAAAGGGAGCGTGGACCTAG<br>R: GCCGCTCGAGTTACTCGGTATTTTTCTTAACGTAGTC
Fig. 2 and Supplementary Fig. 2	
Sth1$_{(1183\text{-}1240)}$ L1204A	F:CAAAAAGAAAAAACCGAAAGCAACCGTTAAAATCAAACTGA ACAAAACCACGGTCCTGG<br>R: CTTTCAGAGCCGTTGATAATTTCAAC
Sth1$_{(1183\text{-}1240)}$ V1206A	F:CAAAAAGAAAAAACCGAAACTGACCGCAAAAATCAAACTGA ACAAAACCACGGTCCTGG<br>R: CTTTCAGAGCCGTTGATAATTTCAAC
Sth1$_{(1183\text{-}1240)}$ I1208A	F:CAAAAAGAAAAAACCGAAACTGACCGTTAAAGCAAAACTGA ACAAAACCACGGTCCTGG<br>R: CTTTCAGAGCCGTTGATAATTTCAAC
Sth1$_{(1183\text{-}1240)}$ L1210A	F:CAAAAAGAAAAAACCGAAACTGACCGTTAAAATCAAAGCAA ACAAAACCACGGTCCTGG<br>R: CTTTCAGAGCCGTTGATAATTTCAAC
Sth1$_{(1183\text{-}1240)}^{M4}$	F:CAAAAAGAAAAAACCGAAAGCAACCGCAAAAGCAAAAGCA AACAAAACCACGGTCCTGG<br>R: CTTTCAGAGCCGTTGATAATTTCAAC
Sth1$_{(1183\text{-}1240)}$ K1207E	F:CAAAAAGAAAAAACCGAAACTGACCGTTGAAATCAAACTGA ACAAAACCACGGTCCTGG<br>R: CTTTCAGAGCCGTTGATAATTTCAAC
Sth1$_{(1183\text{-}1240)}$ K1209E	F:CAAAAAGAAAAAACCGAAACTGACCGTTAAAATCGAACTGA ACAAAACCACGGTCCTGG<br>R: CTTTCAGAGCCGTTGATAATTTCAAC
Taf14$_{(174\text{-}244)}$ F220A	F:GAATAATGTTGAAGAGGGTGAAGCAATAATTGACTTGTATAG TTTACCTGAG<br>R: GTCACGTTCATTTCTGGTGTTTTATTGTC
Taf14$_{(174\text{-}244)}$ F220R	F:GAATAATGTTGAAGAGGGTGAACGTATAATTGACTTGTATAG TTTACCTGAG<br>R: GTCACGTTCATTTCTGGTGTTTTATTGTC
Taf14$_{(174\text{-}244)}$ I222R	F:GAATAATGTTGAAGAGGGTGAATTTATACGTGACTTGTATAG TTTACCTGAG<br>R: GTCACGTTCATTTCTGGTGTTTTATTGTC
Taf14$_{(174\text{-}244\ )}$ F220R/I222R	F:GAATAATGTTGAAGAGGGTGAACGTATACGTGACTTGTATAG TTTACCTGAG<br>R: GTCACGTTCATTTCTGGTGTTTTATTGTC
Sth1^{M4}-gRNA	F1:CGGAATCCTCTTTGAAAAGATAATGTATGATTATGCTTTC<br>R1:GCTCTAAAACAAACTGACCGTCAAGATCAAGATCATTTATC TTTCACTGCGGAGAAG

	F2:ATGATCTTGATCTTGACGGTCAGTTTGTTTTAGAGCTAGAAATAGCAAGTTAAAATAAG<br>R2:AAGGAAAAAAGCGGCCGCAGACATAAAAAACAAAAAAAGCACCACCG
Sth1-TAP	F:TTAAATGAGTTTACTGATGAATGGTTCAAGGAACACTCTTCGTCCATGGAAAAGAGAAG<br>R:GGATATAGTCGTAAAAAAAAAAAACATGTGGTGATGAAAACGTACGACTCACTATAGGG
Taf14-HA	F:ATTGAAAAGTCTATGGGACTACGTTAAGAAAAATACCGAGCGGATCCCCGGGTTAATTAA<br>R:ATACAAACATAAAAGCGCGCATTTAACGCCCTTTTACCTTGAATTCGAGCTCGTTTAAAC
Fig. 3 and Supplementary Fig. 3	
Sth1-pRS313	F: GGACTAGTCACTAGAGTCTTCCTCGTCGC<br>R: CTCGAGGATGCTGAACGAGAATCGCTTC
Taf14-pRS313	F1: CCCCCGGGGGGATTTCTTCTCTAGAATGGCA<br>R1:CGGATGGTTCTTTTTACTGTAGCTACCATGATTAGTTATCT<br>F2:GATAACTAATCATGGTAGCTACAGTAAAAAGAACCATCCG<br>R2: CCCTCGAGGG TCAAACTCATCAAGAGGATTC
Act1	F: TCGTTCCAATTTACGCTGGTT<br>R: CGGCCAAATCGATTCTCAA
Mgm1	F: AAAGTCCGTTGGTGCACTTACA<br>R: TGGCATGTGCTGATCTGTGA
Tir4	F: TGCCGACTACATCACCCTATCC<br>R: GGCATTTGGTCCAAGGAAAA
Yml045w	F: GAAATCCGAGTGATGAGAAGAATGA<br>R: CCGAGCTATAACTTTGGGTTTG
Hlr1	F: GGAGAACCCCTCCACGTATACA<br>R: TCGTCGGCAAAATGACACTTT
Hsp30	F: ACCATGCTACGGACGATGTG<br>R: GCTCTCTGGAACAGCTTCTTTTG
Tps1	F: TTGCACGCCATGGAAGTG<br>R: AACAACCTTGCCCCTCCATT
Gpx1	F: TGCAACGGGCAAAAGCA<br>R: CAGACTTCCCGCTTACTGAATTC
Mst27	F: CAAGGCAGCGGAGATCGA<br>R: GCCGCCTTCGCCAGTAA
Rsm25	F: TGGGCGTGAAAAGGCTGTA<br>R: GCGATCTGAGCCTATCCTCAA
Prs2	F: CCACACCGGAGATCCTTTTG<br>R: TCTTGGAACCCGCATTGC
Exg1	F: CCATCGGTTGGGCTAGAAAC<br>R: CGGCACCATGCAAATCAA
Fks3	F: ACTTGGGTGCGTTGAACCA<br>R: CAACGGGTGTCTCAAGATTTGTAA
Gas2	F: GCGCCTGTTTGGATGAGATT<br>R: TCCAGATTCAGCACCAAAAGG
Gas4	F: GGCTCTTCCGACGTTAGTGAA<br>R: GAGCGCATGCATCTGGATT
Pdc5	F: TTGGGACCACTTGGCCTTATT<br>R: GGTAGCAACTCTGTGGGTTTCG
Ser2	F: TTTGTTATCACCTGCATAGCTCATG<br>R: TCGCAATCTGGTCGATGGT

Fyv5	F: TCTGTCCGAATTTGCTCTCGTA<br>RGCCGAGGAGCTGATCAAG
Pgm2	F: GGTTACGGCCCATCTTTCGT<br>R: GCTGCATATTCGGCAATAATTG
Tps2	F: CACTGTGGGATCTGCATCCA<br>R: GGACTTGCTGAGGATCGGTTAA
Fig. 4 and Supplementary Fig. 4	
INO80$_{(368-390)}$	F: GCGCGGATCCGATTCCAAGTTGAACATCAAAATAAC<br>R:GCCGCTCGAGTTAGTTAATCAGTTTTTCAACCTTTTGAC
Ino80$_{(311-370)}$	F:CGGGATCCATGAACTCGCTGTCTTTAATAACG<br>R: CGCTCGAGTTACTTGGAATCGATTGGATTGTA
Ino80$_{(400-490)}$	F:CGGGATCCGAGGCCTTGAAGAACAACGTTGG<br>R: CGCTCGAGTTATCTTGACATTTTCGTAGAATCC
Ino80$_{(500-550)}$	F:CGGGATCCTCTACAAATTTTAGGAAAACTTC<br>R: CGCTCGAGTTATTCTTCACGCTCATTCTTCTTCC
Ino80$_{EBM}$ L371A	F:GCAAACATCAAAATAACTTTGAAACAG<br>R: CTTGGAATCGATTGGATTG
Ino80$_{EBM}$ I373A	F:GCAAAAATAACTTTGAAACAGTACCAC<br>R: GTTCAACTTGGAATCGATTGGATT
Ino80$_{EBM}$ I375A	F:GCAACTTTGAAACAGTACCACGTC<br>R: TTTGATGTTCAACTTGGAATC
Ino80$_{EBM}$ L377A	F:GCAAAACAGTACCACGTCAAAAGGTT<br>R: AGTTATTTTGATGTTCAACTTGGAA
Ino80$_{EBM}^{M4}$	F:GCAAACGCAAAAGCAACTGCAAAACAGTACCACGTCAAAAG<br>GTT<br>R: CTTGGAATCGATTGGATTG
Sas3$_{(111-129)}$	F:GCGCGGATCCAGCGAGGAGTTGAAGGTTAGAATTAAG<br>R:GCCGCTCGAGTTATTCAAAATTGAAAAATTTGATAGAATC
Sas3$_{(96-110)}$	F:CGGGATCCATGGGAGCAGTAAGTTTCCCCATTATTGAACCTA<br>ATATTGAAGTC<br>R:CGCTCGAGTTAGACTTCAATATTAGGTTCAATAATGGGGAAA<br>CTTACTGCTCCCAT
Snf5$_{(771-800)}$	F:CGGGATCCGCCAGATAGACCTAAGCCAGTT<br>R: CGCTCGAGTTACTCTTCTTTTGTATTAACTTTCC
Snf5$_{(700-800)}$	F:CGGGATCCACACAGTAGCAGCAGGAAATGC<br>R: CGCTCGAGTTACTCTTCTTTTGTATTAACTTTCCC
Snf5$_{(800-905)}$	F:CGGGATCCGAGTTCGCAGCAGCGCCCAATG<br>R:CGCTCGAGTTACTATGTATTCTGTGTGTTATTGTTACTGC
Snf5$_{(700-741)}$	F:CGGGATCCACACAGTAGCAGCAGGAAATGC<br>R: CGCTCGAGTTAACCAGGCATTAAAGTGCTAGG
Snf5$_{(741-771)}$	F:CGGGATCCGGTGGTGTTGACGTAGGCCCTTC<br>R: CGCTCGAGTTATGAAACTGGCTTAGGTCTATCTGGCCT
Snf5$_{EBM}$ L785A	F:GCACTACTTTCTATTAAACTGCCTG<br>R: CGAATGACCCGGAATATGG
Snf5$_{EBM}$ L787A	F: GCATCTATTAAACTGCCTGGGAAAG<br>R: TAGTAGCGAATGACCCGGAATATG
Snf5$_{EBM}$ I789A	F:GCAAAACTGCCTGGGAAAGTTAATAC<br>R: AGAAAGTAGTAGCGAATGACCCGG
Snf5$_{EBM}$ L791A	F:GCACCTGGGAAAGTTAATACAAAAGA<br>R: TTTAATAGAAAGTAGTAGCGAATGAC
Snf5$_{EBM}^{M4}$	F:GCACTAGCATCTGCAAAAGCACCTGGGAAAGTTAA<br>R: CGAATGACCCGGAATATGGTC
Taf2$_{(1393-1406)}$	F:CGGGATCCTCAAGATCGTTTATGGTTAAGATAAGAACAAAGA

	ATGATGCTAAG<br>R:CGCTCGAGTAACTTAGCATCATTCTTTGTTCTTATCTTAACCA TAAACGATCTTGA
Tfg1$_{(580-605)}$	F:CGGGATCCGACACTCTATCCAAATCCAAGAGATCTTCTCCAA AGAAACAGCAAAAGAAAGCTACAAATGCGCATGTGCATAAAG AG<br>R:CGCTCGAGTTACTCTTTATGCACATGCGCATTTGTAGCTTTCT TTTGCTGTTTCTTTGGAGAAGATCTCTTGGATTTGGATAGAGTG TC
Tfg1$_{(605-629)}$	F:CGGGATCCGAGCCAACTTTGAGGGTGAAAAGTATTAAAAACT GTGTCATTATCTTGAAGGGGGATAAGAAAATACTGAAAAGC<br>R:CGCTCGAGTTAGCTTTTCAGTATTTTCTTATCCCCCTTCAAGA TAATGACACAGTTTTTAATACTTTTCACCCTCAAAGTTGGCTC
Tfg1$_{(629-679)}$	F:CGGGATCCAGCTTCCCAGAGGGAGAA<br>R: CGCTCGAGTTATGTTATTGTAGGCGCTGGAGTTTC
Tfg1$_{(580-735)}$	F: GCGCGGATCCGACACTCTATCCAAATCCAAGAGATC<br>R: GCGCCTCGAGTTACTCTTTCTTTAATTCCATGTGGTC
Tfg1$_{(680-735)}$	F: GCGC GGATCC GAAAAAGATATCATCGAGGCTATTG<br>R: GCGCCTCGAGTTACTCTTTCTTTAATTCCATGTGGTC
Fig. 5 and Supplementary Fig. 5	
Sth1$_{(1183-1240)}$-RFP	F1:CGGGATCCGAAGTAAAAAGTAGCTCCGTTG<br>R1:CCTCGGAGGAGGCTTTGGCGTCAGTCTTCTTCGCG<br>F2:GACTGCAGCCAAAGCCTCCTCCGAGGACGTCATC<br>R2:CCGCTCGAGTTAGGCGCCGGTGGAGTGGC
Snf5$_{(771-800)}$-CFP	F1:ATGTCGGACTCAGAAGTCAATC<br>R1:AAAAGTTCTTCTCCTTTGCTCTCTTCTTTTGTATTAACTTTCC CAGGC<br>F2:AAGTTAATACAAAAGAAGAGAGCAAAGGAGAAGAACTTTTC AC<br>R2:CCGCTCGAGTTATTTGTAGAGCTCATCCATGCCA
Taf14$_{(174-244)}$-GFP	F:CGGGATCCAAAGGGACGGTGGACCTAGAA<br>R:CCGCTCGAGCTCGGTATTTTTCTTAACGTAGTCC
Fig. 6	
Bdf1$_{(525-595)}$	F: GCGCGGATCCAAAACAGTAGTGACATATGATATG<br>R: GCCGCTCGAGTTAGGATGAGCTTTCATATTGTCTAAAG
Bdf2$_{(514-585)}$	F: GCGC GGATCC AAGCATTCAGTTGACGATTTAAAG<br>R:GCCGCTCGAGTTAGTTATTTTTTTCTCAAAATATCTCTC
AF9($_{(490-568)}$	F: CGGGATCCTCAGATAAGCAAATAAAGAATGGTG<br>R: CCGCTCGAGTCAGGATGTTCCAGATGTTTCC
AF9$_{(490-568)}$-GFP	F: CGGGATCCTCAGATAAGCAAATAAAGAATGGTG<br>R: CCGCTCGAGTCAGGATGTTCCAGATGTTTCC
BRD$_{(601-683)}$-GFP	F: CGGGATCCTCGGAGGAAGAGGACAAGTGC<br>R: CCGCTCGAGAGCTTGAGGTTTCCTTTTCTTCCG
