# Supplementary material for: Taf14 recognizes a common motif in transcriptional machineries and facilitates their clustering by phase separation
Source: Nat Commun. 2020 Aug 21;11:4206. doi: 10.1038/s41467-020-18021-7 (PMC7442819; doi:10.1038/s41467-020-18021-7)
Supplement: Supplementary file 3 — Reporting summary [file 41467_2020_18021_MOESM3_ESM.pdf]

nature research

Corresponding author(s): Yong Chen

Last updated by author(s): Jul 26, 2020

Reporting Summary

Nature Research wishes to improve the reproducibility of the work that we publish. This form provides structure for consistency and transparency in reporting. For further information on Nature Research policies, see Authors & Referees and the Editorial Policy Checklist .

Statistics

For all statistical analyses, confirm that the following items are present in the figure legend, table legend, main text, or Methods section.

n/a	Confirmed	
☐	☒	The exact sample size (*n*) for each experimental group/condition, given as a discrete number and unit of measurement
☐	☒	A statement on whether measurements were taken from distinct samples or whether the same sample was measured repeatedly
☐	☒	The statistical test(s) used AND whether they are one- or two-sided<br>*Only common tests should be described solely by name; describe more complex techniques in the Methods section.*
☒	☐	A description of all covariates tested
☒	☐	A description of any assumptions or corrections, such as tests of normality and adjustment for multiple comparisons
☐	☒	A full description of the statistical parameters including central tendency (e.g. means) or other basic estimates (e.g. regression coefficient) AND variation (e.g. standard deviation) or associated estimates of uncertainty (e.g. confidence intervals)
☐	☒	For null hypothesis testing, the test statistic (e.g. *F*, *t*, *r*) with confidence intervals, effect sizes, degrees of freedom and *P* value noted<br>*Give P values as exact values whenever suitable.*
☒	☐	For Bayesian analysis, information on the choice of priors and Markov chain Monte Carlo settings
☒	☐	For hierarchical and complex designs, identification of the appropriate level for tests and full reporting of outcomes
☒	☐	Estimates of effect sizes (e.g. Cohen's *d*, Pearson's *r*), indicating how they were calculated

Our web collection on statistics for biologists contains articles on many of the points above.

Software and code

Policy information about availability of computer code

Data collection	Gel images were taken by ImageQuant LAS4000. Fluorescent images were taken by a Zeiss LSM 710 microscope. ITC assays were performed using a MicroCal ITC200 system (GE Healthcare). Circular dichroism spectra were collected on a Chirascan v100 spectrometer (AppliedPhotophysics). All NMR spectra were collected on Agilent DD2 600 MHz spectrometer equipped with a cryogenic probe or DD2 700 MHz spectrometer equipped with a HCN z-gradient room temperature probe.
Data analysis	Origin 7.0 , DAVID v6.7, PyMOL 1.5, GraphPad Prism 8.0, NMR: NMRPipe 9.7, SPARKY 3.115, CYANA 2.1, PSVS 1.5, CNS 1.2

For manuscripts utilizing custom algorithms or software that are central to the research but not yet described in published literature, software must be made available to editors/reviewers. We strongly encourage code deposition in a community repository (e.g. GitHub). See the Nature Research guidelines for submitting code & software for further information.

Data

Policy information about availability of data

All manuscripts must include a data availability statement. This statement should provide the following information, where applicable:

- Accession codes, unique identifiers, or web links for publicly available datasets
- A list of figures that have associated raw data
- A description of any restrictions on data availability

The chemical shifts have been submitted to the BMRB (accession code 36309), and the structure ensemble and NOE restraint file have been submitted to deposited in the PDB with (accession code 6LQZ). RNA-seq data are available in the SRA database under an accession code SRP238570. The source data underlying Figs 2e, 3e, 5d, 5e and Supplementary Figs 2e, 2f, 3d, 3e, 4a-c, 4f are provided as a Source Data file. Other data are available from the corresponding author upon reasonable request.

Field-specific reporting

Please select the one below that is the best fit for your research. If you are not sure, read the appropriate sections before making your selection.

☒ Life sciences ☐ Behavioural & social sciences ☐ Ecological, evolutionary & environmental sciences

For a reference copy of the document with all sections, see nature.com/documents/nr-reporting-summary-flat.pdf

Life sciences study design

All studies must disclose on these points even when the disclosure is negative.

Sample size	NA
Data exclusions	NA
Replication	NA
Randomization	NA
Blinding	NA

Reporting for specific materials, systems and methods

We require information from authors about some types of materials, experimental systems and methods used in many studies. Here, indicate whether each material, system or method listed is relevant to your study. If you are not sure if a list item applies to your research, read the appropriate section before selecting a response.

Materials & experimental systems

n/a	Involved in the study	
☐	☒	Antibodies
☐	☒	Eukaryotic cell lines
☒	☐	Palaeontology
☒	☐	Animals and other organisms
☒	☐	Human research participants
☒	☐	Clinical data

Methods

n/a	Involved in the study	
☒	☐	ChIP-seq
☒	☐	Flow cytometry
☒	☐	MRI-based neuroimaging

Antibodies

Antibodies used	Anti-HA antibodyl(Cell Signaling 3724s), Anti-TAP antibody (Invitrogen, CAB1001), HRP secondary antibody (Jackson ImmunoResearch, 111-035-003)
Validation	Cell Signaling 3724s: HA-Tag (C29F4) Rabbit mAb detects exogenously expressed proteins containing the HA epitope tag. It is a monoclonal antibody is produced by immunizing animals with a synthetic peptide containing the influenza hemagglutinin epitope (YPYDVPDYA). Species Reactivity:All Species Expected. Application: ChIP, IP, WB, DB, ICC, IF, IHC .<br>CAB1001 is polyclonal primary antibody detecting TAP. Host: Rabbit. CAB1001 has been successfully used in Western Blot, IP, and ChIP applications. The CAB1001 immunogen is a KLH conjugated peptide representing the C-terminus of the TAP construct after TEV cleavage. Reactivity: Saccharomyces cerevisiae, Homo sapiens, Schizosaccharomyces pombe, Candida albicans,

Eukaryotic cell lines

Policy information about cell lines

Cell line source(s)	BY4742 yeast strain from Jinqiu Zhou lab
Authentication	*Describe the authentication procedures for each cell line used OR declare that none of the cell lines used were authenticated*
Mycoplasma contamination	*Confirm that all cell lines tested negative for mycoplasma contamination OR describe the results of the testing for mycoplasma contamination OR declare that the cell lines were not tested for mycoplasma contamination*
Commonly misidentified lines (See ICLAC register)	*Name any commonly misidentified cell lines used in the study and provide a rationale for their use*
